# Patterns of immune cell infiltration and oxidative stress in cervical cancer

**DOI:** 10.3389/or.2025.1570071

**Published:** 2025-09-11

**Authors:** Andrea Mlambo, Shuyue Su, Qhaweni Dhlamini, Yuyang Zhang

**Affiliations:** ^1^ The First School of Medicine, School of Information and Engineering, Wenzhou Medical University, Wenzhou, China; ^2^ Department of Pulmonary and Critical Care Medicine, The Quzhou Affiliated Hospital of Wenzhou Medical University, Quzhou People’s Hospital, Quzhou, Zhejiang, China; ^3^ Department of Gynecology, The First Affiliated Hospital of Wenzhou Medical University, Wenzhou, China

**Keywords:** tumor microenvironment, oxidative stress, immune cell infiltration, HPV oncogenes, therapeutic strategies, cervical cancer

## Abstract

Cervical cancer (CeCa) remains a significant global health burden, with complex interactions between oxidative stress and immune response playing critical roles in its pathogenesis and progression. This review synthesizes current knowledge on the molecular mechanisms linking oxidative stress pathways and immune evasion, particularly focusing on human papillomavirus oncogenes *E6* and *E7*. We highlight the dual roles of immune components such as Type 17 T helper (Th17) cells and the antioxidant enzyme superoxide dismutase 2 (SOD2), which exhibit context-dependent tumor-promoting and suppressive functions. While extensive mechanistic insights have been gained, translation to clinical practice remains limited, partly due to inconsistent biomarkers and incomplete understanding of therapeutic resistance. Recent advances in targeted therapies, including mitochondrial inhibitors, Immune checkpoint inhibitors (ICIs) (e.g., pembrolizumab, nivolumab), and *PARP* inhibitors, demonstrate promise but face translational hurdles such as assay variability and immune-related adverse events. Future research must address gaps including predictive biomarker development, noninvasive monitoring via liquid biopsy, and rational combination therapies integrating redox modulation and immunotherapy. Enhanced multi-omics integration and refined preclinical models are essential to advance personalized treatment strategies for CeCa.

## 1 Introduction

Cervical cancer (CeCa), despite being largely preventable, remains a leading cause of cancer-related mortality among women worldwide. In 2022, an estimated 662,301 new cases and 348,874 deaths were reported globally (1, 2). According to the American Cancer Society, while long-term declines in the US have plateaued, there are notable age-specific differences. Incidence has increased by about 1.7% per year among women 30–44 years old from 2012 to 2019, while it has declined by approximately 11% per year among those 20–24 years old (3, 4).

In China, CeCa is a significant concern, accounting for 150,659 new cases and 55,694 deaths in 2022, which represents roughly 23% of global incidents and 16% of global deaths (1). The situation is further compounded by urban-rural disparities, inadequate screening, and uneven HPV vaccine uptake (5).

**FIGURE 1:**
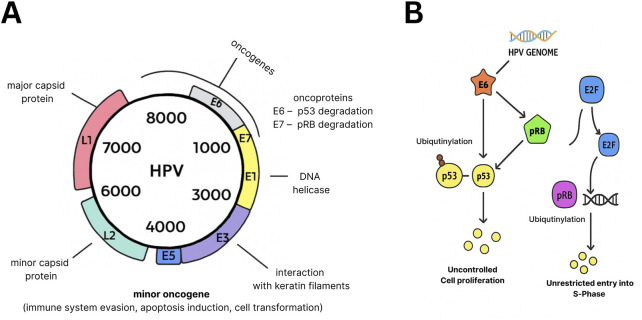
Oncogenic functions of HPV E6 and E7. **(A)** Genomic organization of the HPV genome, showing capsid proteins (L1, L2) and early genes (E1–E7). **(B)** Mechanisms of oncogenesis: E6 promotes ubiquitination and degradation of p53, while E7 inactivates pRb to release E2F, driving S-phase entry and uncontrolled proliferation. Both proteins additionally facilitate immune evasion through downregulation of MHC-I and disruption of interferon signaling (not shown).

The etiology of CeCa is strongly linked to persistent infection with high-risk human papillomavirus (HPV) types, especially human papillomavirus type 16 (HPV16) and human papillomavirus 18 (HPV18). The viral oncogenes E6 and E7 disrupt tumor suppressor pathways by inactivating p53 and retinoblastoma protein (pRb), facilitating uncontrolled cellular proliferation and tumor progression (6–8). Beyond oncogenic transformation, HPV E6/E7 also contribute to immune evasion by modulating antigen presentation and dampening immune surveillance, creating an immunosuppressive microenvironment conducive to tumor growth [(9); Figure 1A].

Oxidative stress, characterized by an imbalance between reactive oxygen species (ROS) generation and antioxidant defenses, has emerged as a critical factor in cervical carcinogenesis. Excess ROS can induce DNA damage, lipid peroxidation, and protein oxidation, promoting genetic instability and oncogenic signaling (2). Interestingly, oxidative stress plays a dual role by also activating immune responses, which can either suppress or promote tumor progression depending on the context. The crosstalk between oxidative stress pathways and immune regulation in CeCa is complex and incompletely understood, necessitating further exploration (10).

The tumor microenvironment (TME) in CeCa includes diverse immune cells, such as tumor-associated macrophages (TAMs), T cells, and regulatory T cells (T_regs_), which influence cancer development and response to therapy. Immune evasion mechanisms mediated by HPV, coupled with oxidative stress-induced inflammation, contribute to TME remodeling and tumor immune escape (2). However, conflicting evidence exists regarding the role of certain immune subsets, such as Type 17 T helper (Th17) cells, which have been reported to exhibit both pro- and anti-tumorigenic effects in different studies (10). This highlights the need for more detailed investigation into immune dynamics within CeCa.

## 2 Core pathogenesis: HPV–immunity–redox crosstalk

### 2.1 HPV-mediated immune evasion mechanisms

**FIGURE 2:**
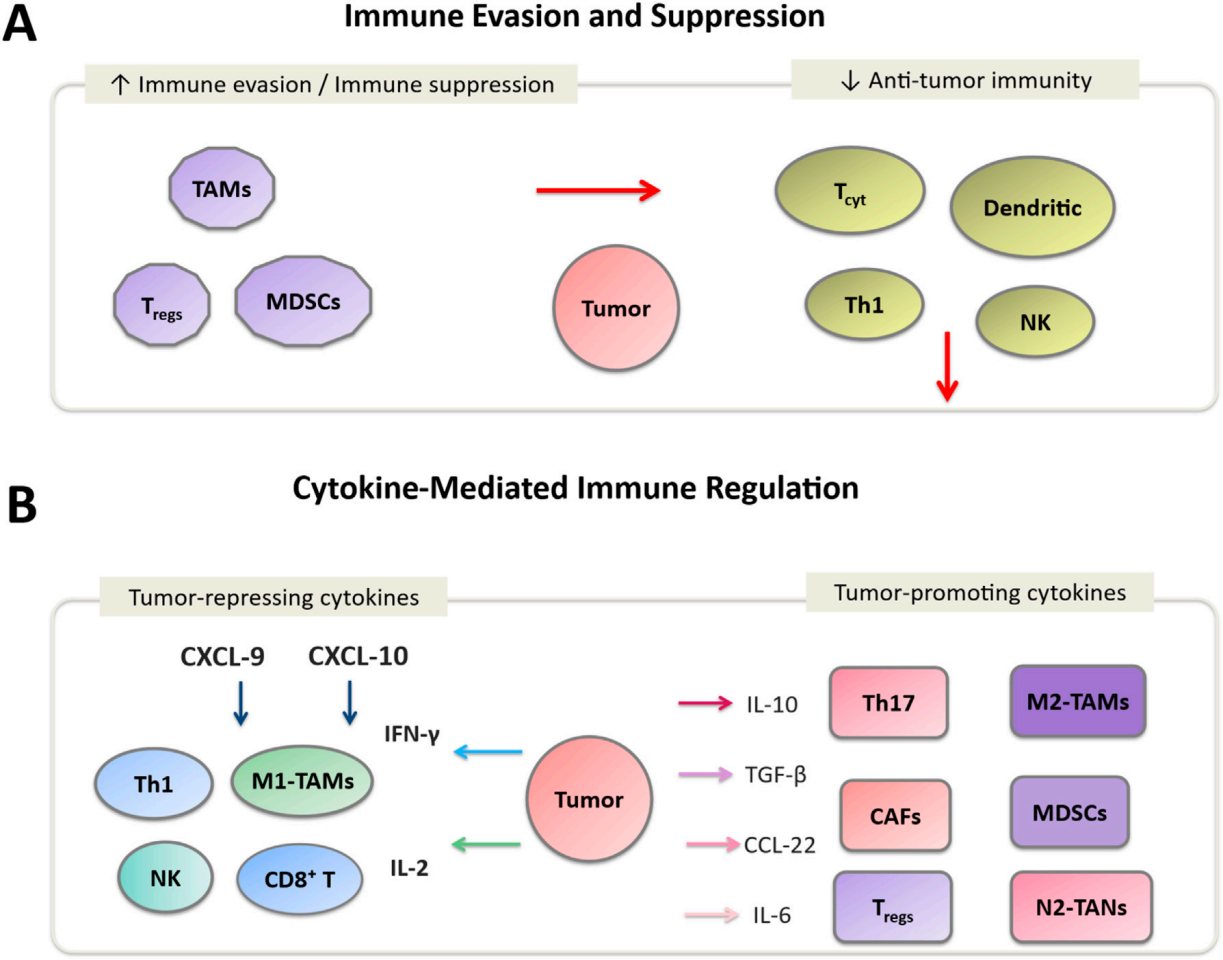
Immune regulation in the cervical cancer tumor microenvironment (TME). **(A)** Immune evasion mechanisms involving Tregs, MDSCs, and M2-TAMs, along with immunosuppressive cytokines (IL-10, TGF-β, IL-6) that inhibit CD8^+^ T cells and NK cells. **(B)** Cytokine-mediated modulation: tumor-repressing cytokines (IFN-γ, IL-2, CXCL-9) activate effector immune cells (CD8^+^ T cells, NK cells, M1-TAMs), while tumor-promoting cytokines (IL-10, TGF-β, IL-6) enhance angiogenesis, immune escape, and tumor progression.

Persistent infection with high-risk HPV types drives cervical carcinogenesis through coordinated immune-evasion strategies. The viral oncoproteins E6 and E7 impair antigen presentation by downregulating major histocompatibility complex class I (MHC-I) molecules on infected cell surfaces, limiting recognition by CD8^+^ T cells (9). Furthermore, these oncogenes interfere with interferon (IFN) signaling pathways, reducing the production of type I IFNs that are crucial for antiviral immunity (11). This suppression of innate immune responses fosters an immunosuppressive TME that promotes viral persistence and tumor progression [(12), Figure 2A].

Innate immunity disruption: HPV employs multiple strategies to evade innate immune responses. β-HPV type 38 E6/E7 proteins have been shown to downregulate Toll-like receptor 9 (TLR9), a DNA sensor important for antiviral responses, in keratinocyte models (13, 14). While these findings are specific to HPV38 and should not be directly generalized to high-risk mucosal types such as HPV16/18, some evidence suggests similar mechanisms may occur. Cell-based experiments also demonstrate that HPV16 E6 interferes with the RIG-I pathway by binding the ubiquitin ligase TRIM25 (15, 16), causing TRIM25 degradation and preventing RIG-I activation via K63-linked ubiquitination (16). The impaired RIG-I pathway further suppresses downstream type I interferon signaling *in vitro* and requires clinical confirmation in CeCa patients (17–19).

Adaptive immunity suppression: HPV oncoproteins target the cGAS–STING DNA-sensing pathway (20). HPV16/18 oncoproteins downregulate STING transcription in experimental systems (21). This is in line with the lower level of STIING mRNA in HPV-positive cervical lesions compared with normal tissue. These observations are most consistent with transcriptional downregulation by viral proteins rather than established epigenetic silencing in patients (22, 23).

The immune escape facilitated by HPV oncogenes also involves modulation of immune checkpoint molecules such as programmed death ligand-1 (PD-L1), which further inhibits T-cell activation (24, 25). Elevated PD-L1 expression has been observed in CeCa tissues and correlates with poor prognosis and resistance to conventional therapies (26–28).

Clinically, durable responses to anti-PD-1 therapy have been observed in the cervical cancer cohort of the multi-cohort KEYNOTE-158 study, particularly in PD-L1–positive disease (29, 30). HPV oncogenes additionally induce chronic inflammation via COX-2 and PGE2, activating the COX–PG pathway (31, 32).

### 2.2 HPV-induced oxidative stress

Persistent HPV infection disrupts cellular redox balance through coordinated actions of viral oncoproteins (33). E6 degrades p53, impairing transcriptional activation of antioxidant genes (34, 35), while E7 inactivates pRb, promoting cell-cycle progression under oxidative conditions (36–38). This dual assault creates a pro-oxidant state characterized by mitochondrial dysfunction, in which damaged electron-transport chains generate excessive reactive oxygen species (ROS) (6, 33, 39).

Metabolic reprogramming: HPV further amplifies oxidative stress through metabolic changes. The E4 protein can disrupt mitochondrial–cytoskeletal interactions, reducing membrane potential and promoting apoptosis resistance (40, 41). While E6/E7 upregulate glycolytic enzymes to sustain the Warburg effect (42, 43). This metabolic shift creates a feed-forward loop in which mitochondrial ROS generation perpetuates further oxidative damage (40, 44, 45).

DNA damage and mutagenesis: The resulting oxidative stress induces nuclear and mitochondrial DNA damage, including mutagenic 8-hydroxy-2′-deoxyguanosine (8-OHdG) lesions that contribute to oncogenic transformation. The resulting oxidative stress induces nuclear and mitochondrial DNA damage, including mutagenic 8-hydroxy-2′-deoxyguanosine (8-OHdG) lesions that drive oncogenic transformation (8, 43, 46). These immunosuppressive networks further correlate with both TAM polarization states and advanced disease progression (43, 47). Certain mitochondrial DNA polymorphisms (e.g., C150T) may also exacerbate this process, increasing susceptibility to HPV persistence and cervical cancer development (48).

### 2.3 Context-dependent immune modulation

The TME in CeCa comprises diverse immune cells that influence tumor development and therapy response (Table 1). Conflicting evidence exists regarding Th17 cells: in early lesions Th17-associated IL-17 can participate in neutrophil recruitment (49).Whereas in advanced disease Th17 cells have been linked to angiogenesis, immunosuppression, and tumor progression (50, 51). Similarly, the antioxidant enzyme superoxide dismutase 2 (SOD2) demonstrates context-dependent functions, acting as both protective and tumor-supportive depending on redox status and treatment exposure (52, 53).

**TABLE 1:** Immune cell subsets in cervical cancer: compartmental distribution, stage-specific dynamics, clinical correlations, and direction of prognostic effect.

Subset	Compartment	Typical observation	Clinical readout	Direction
CD8^+^ T cells	Tumor nests vs. stroma	Intratumoral > peritumoral density	OS/PFS/response to ICI	Intratumoral ↑ → better
Tregs	Peritumoral stroma	Enriched; high Treg:CD8	OS/PFS/ICI response	High ↑ → worse
TAMs (M2-skewed)	Stroma and nests	CD163^+^/Arg1^+^ enrichment	Stage/LN^+^/OS	High ↑ → worse
Th17 (blood)	Peripheral blood	↑ Th17/Treg in CIN; ↑ with stage	Stage/LN^+^/response	Higher ↑ → worse (advanced)
Th17 (tissue)	Tumor tissue (TILs)	↑ with stage; heterogeneity	OS/PFS	Usually worse; squamous paradox

## 3 Immune landscape in cervical cancer

The cervical cancer tumor microenvironment (TME) is composed of diverse immune populations whose density, phenotype, and spatial organization influence prognosis and therapeutic response (Figure 1B). The balance between effector and suppressive subsets, as well as their localization within tumor nests, peritumoral stroma, or circulation, critically shapes clinical outcomes.

### 3.1 Cytotoxic T cells (CD8^+^)

CD8^+^ tumor-infiltrating lymphocytes (TILs) constitute a principal effector population in antitumor immunity (54). Their distribution within the TME has prognostic significance: high intratumoral (nest) density is generally associated with favorable outcomes, whereas exclusion to the peritumoral stroma correlates with immune escape and resistance to immune checkpoint inhibitors (ICIs) (55, 56). Spatial metrics, such as proximity to the tumor–stroma interface, are increasingly recognized as key determinants of therapeutic efficacy and should be systematically reported in clinical studies (57). High intratumoral CD8^+^ density has been shown to be an independent predictor of improved progression-free and overall survival in cervical cancer patients (58). Meta-analyses also confirm the prognostic value of CD8^+^ TILs, with increased infiltration correlating with significantly better survival outcomes across cervical cohorts (59).

### 3.2 Regulatory T cells (T_regs_)

Regulatory T cells (T_regs_) are enriched in the peritumoral stroma of cervical tumors, where they exert suppressive effects on effector T cells via cytotoxic T-lymphocyte–associated protein 4 (CTLA-4) signaling and secretion of inhibitory cytokines such as interleukin-10 (IL-10) and TGF-β: Transforming Growth Factor-β (Figure 1B). High _*Treg*_ density and elevated T_reg_:CD8 ratios are consistently associated with poor prognosis and diminished ICI efficacy. This reflects the creation of an exclusionary stromal niche that is unfavorable for effective antitumor immunity (47, 60).

### 3.3 Tumor-associated macrophages (TAMs)

Cervical tumors frequently exhibit polarization toward an M2-skewed macrophage phenotype (61). These tumor-associated macrophages (TAMs) are characterized by arginase-1 expression, pro-angiogenic activity, and suppression of T-cell effector function [(62, 63), Figure 2B]. Enrichment of M2-like TAMs correlates with advanced disease stage, lymph-node metastasis, and resistance to therapy (64–66). Accurate characterization requires specification of phenotypic markers such as CD68/CD163 and careful annotation of their anatomical compartment within tumor nests or stroma (67).

### 3.4 Th17 cells—stage- and compartment-specific roles

T helper 17 (Th17) cells, defined by interleukin-17 (IL-17) secretion, display stage- and compartment-specific behaviors in CeCa.

Early disease (CIN/early tumors): Peripheral blood studies demonstrate an elevated Th17: *T*_*reg*_ ratio compared with healthy controls, reflecting early immune dysregulation. This imbalance persists into invasive disease, though without substantial systemic amplification (68, 69).

Advanced disease: Th17 accumulation in blood and tumor tissue correlates with higher clinical stage, lymph-node metastasis, and poor outcomes (68, 69). Increased Th17 frequencies under treatment pressure (e.g., chemoradiation) have been linked to poor therapeutic response and early relapse (70).

Notably, data remain heterogeneous. In squamous histologies, higher intratumoral IL-17^+^ cell density has been associated with improved survival (69), though much of this IL-17 signal has been derived from neutrophils rather than Th17 cells (71, 72). Thus, IL-17 alone cannot be employed as surrogate marker for Th17 activity. The impact of Th17 cells depends on the histological subtype of the tumor. In squamous cell carcinoma, Th17 responses may provide some protective or beneficial effects, whereas in adenocarcinoma they are mostly harmful and contribute to disease progression. In select niches, Th17 cells may also indirectly promote antitumor immunity by recruiting cytotoxic effector cells (69).

Overall, the evidence supports a pro-tumorigenic role for Th17 cells in advanced cervical cancer, while recognizing histological and spatial exceptions.

## 4 Biomarkers: evidence levels and clinical utility

Biomarkers reflecting oxidative stress and immune contexture are increasingly investigated in context of CeCa. While several biomarkers demonstrate biological and prognostic relevance, most remain exploratory due to methodological variability and lack of clinical validation. A tabulated summary of these methods can be found in Table 2.

**TABLE 2:** Oxidative stress and immune biomarkers in cervical cancer: analytical platforms, evidence levels, clinical status, and key limitations.

Biomarker	Analytical Platform(s)	Evidence level	Clinical status	Key limitations
8-hydroxy-2′-deoxyguanosine (8-OHdG)	ELISA; LC-MS/MS	Investigational; correlates with progression and TAM polarization	Not clinically validated in CeCa	Variability; cross-reactivity; lack of standards; oxidation/storage issues
Cell-free mitochondrial DNA (cf-mtDNA)	qPCR/digital PCR; NGS	Exploratory; small cohorts link to treatment response	Early stage; not validated	Sensitive to tube type, processing delay, freeze–thaw cycles
PD-L1 expression	IHC (e.g., 22C3 pharmDx, SP263)	Predictive; supported by KEYNOTE-158/826 trials	FDA-approved for pembrolizumab	Expression heterogeneity; assay and spatial variability
Tumor-infiltrating lymphocytes (CD8^+^ TILs)	IHC; multiplex IF; digital pathology	Prognostic; high CD8^+^ density → better OS/PFS	Investigational in CeCa	No standardized scoring; spatial context and assay variability

Abbreviations. Ceca, Cervical Cancer; ELISA, Enzyme-Linked Immunosorbent Assay; LC-MS/MS, Liquid Chromatography Tandem Mass Spectrometry; qPCR, quantitative polymerase chain reaction.

NGS, Next-Generation Sequencing; TAM, Tumor-Associated Macrophage; IHC, immunohistochemistry.

IF, Immunofluorescence; OS, Overall Survival; PFS, Progression-Free Survival; FDA, food and drug administration.

### 4.1 Oxidative stress biomarkers

Among oxidative stress–related biomarkers, 8-hydroxy-2′-deoxyguanosine (8-OHdG) is the most widely studied DNA lesion. It reflects ROS-mediated oxidative damage and has been linked to genomic instability, carcinogenesis, and macrophage polarization (8, 43, 46). Detection methods include enzyme-linked immunosorbent assay (ELISA) and liquid chromatography–tandem mass spectrometry (LC-MS/MS). However, these platforms are not directly comparable, as pre-analytical oxidation, sample storage, and assay-specific cross-reactivity introduce variability. Such inter-assay inconsistency currently precludes standardized application in clinical practice (73, 74). Thus, 8-OHdG should therefore be regarded as investigational, with evidence best interpreted as correlational rather than as a validated decision-making tool.

Circulating cell-free mitochondrial DNA (cf-mtDNA) represents another emerging marker (75). Its potential utility lies in real-time monitoring of treatment response and disease dynamics. Yet cf-mtDNA quantification is also highly sensitive to technical variables, including collection tube type, processing delays, freeze–thaw cycles, extraction chemistry, and amplicon length. Readers are directed to (75) for a detailed review of these quantitative methods (75). Early cohort studies suggest that cf-mtDNA levels may reflect disease activity, but reproducibility challenges underscore its current exploratory status, pending prospective validation (73, 74).

### 4.2 Immune biomarkers

PD-L1 expression serves as a clinically established predictive biomarker in CeCa, guiding patient selection for immune checkpoint inhibitor (ICI) therapy (76, 77). Clinical benefit has been demonstrated in the CeCa cohort of KEYNOTE-158, though responses occur in only a subset of PD-L1–positive patients (78). PD-L1 utility is constrained by intratumoral heterogeneity, assay variability, and temporal dynamics of expression, which limit its standalone predictive accuracy (78). Consequently, multiplexed or composite biomarker strategies are increasingly advocated.

Tumor-infiltrating lymphocytes (TILs), particularly CD8^+^ T cells, do not also retain universally consistent prognostic value across studies and different types of cancers (79). Higher intratumoral CD8^+^ density is associated with improved overall survival (OS) and progression-free survival (PFS), reflecting a more favorable immune contexture (80–82). To maximize reproducibility, standardized immunohistochemistry or multiplex immunofluorescence assays with attention to spatial localization (tumor nests vs. stroma) are recommended (47, 83).

## 5 Therapeutics: current standards and emerging combinations

The therapeutic landscape of CeCa has evolved with the advent of immune checkpoint inhibitors (ICIs, e.g., PDL-1) and exploratory redox-targeting agents. However, clinical benefit remains heterogeneous, underscoring the need for biomarker-guided selection and rational combinations.

### 5.1 Current standards: immune checkpoint inhibitors

Immune checkpoint blockade has transformed the management of recurrent and metastatic CeCa, though responses remain confined to subsets of patients.

The KEYNOTE-158 trial established pembrolizumab as a therapeutic option in recurrent/metastatic CeCa. In the dedicated cervical cohort (n:98), the overall response rate (ORR) was 12.2%, with a modest increase to 14.6% among PD-L1–positive tumors. Responses were not frequent in PD-L1–negative disease. While durable responses were achieved in some patients, most non-responders progressed, reflecting the persistence of an immunosuppressive TME. Immune-related adverse events occurred in ∼12% of patients, consistent with the broader safety profile of pembrolizumab (84).

The phase III KEYNOTE-826 trial redefined the standard of care for persistent, recurrent, or metastatic disease. Pembrolizumab combined with platinum-based chemotherapy ± bevacizumab significantly improved both overall survival (OS) and progression-free survival (PFS). At 22 months of median follow-up, OS reached 24.4 months compared with 16.5 months in the control arm, with a hazard ratio for death of 0.64 (95% CI 0.50–0.81) in PD-L1–positive patients. These results established pembrolizumab plus chemotherapy (with or without bevacizumab) as the global first-line standard for PD-L1–positive advanced CeCa (85).

### 5.2 Other immunotherapeutic approaches

Therapeutic HPV vaccines represent an area of ongoing development. The ISA101 peptide vaccine combined with nivolumab demonstrated an ORR of ∼33% in a cohort of HPV16-positive solid tumors, predominantly head-and-neck squamous cell carcinoma. Only a minority of patients had CeCa, limiting generalizability of the results (86). Additional vaccine modalities, including DNA- and viral vector–based platforms, are under early-phase evaluation, but no vaccine has yet achieved regulatory approval forCeCa.

### 5.3 Redox-targeting agents and repurposed drugs

Given the role of oxidative stress in HPV-driven carcinogenesis, redox modulators are under investigation. BMX-001, a manganese porphyrin radiomodulator, has entered clinical trials primarily in glioblastoma and head-and-neck cancer (87). Its application in CeCa remains investigational with no disease-specific data available (39).

Mdivi-1, a mitochondrial division inhibitor widely employed in preclinical studies, has demonstrated mechanistic utility but possesses off-target effects precluding clinical suitability (88, 89, 102).

Drug repurposing represents an additional therapeutic avenue. For instance, Metformin has exhibited antiproliferative and redox-modulating effects in CeCa cell lines and xenograft models (90, 91), though randomized clinical evidence in CeCa is lacking. Similarly, imipramine and nelfinavir have shown ROS-modulating and anti-HPV effects in preclinical models (92, 93), with early-phase trials underway in other malignancies (Table 3). However, cervical-specific clinical validation remains absent, which positions these agents as hypothesis-generating rather than clinically actionable (94, 95).

**TABLE 3:** Therapeutic landscape in advanced cervical cancer: mechanisms, clinical trial phases, endpoints, and outcomes of current and investigational agents.

Agent/Approach	Mechanism	Phase/Trial	Population	End points	Outcome/Status
Pembrolizumab (KEYNOTE-158)	PD-1 blockade	Phase II (multi-cohort)	R/M CeCa, n = 98	ORR, safety	ORR 12.2% overall; 14.6% PD-L1^+^; durable, manageable irAEs
Pembrolizumab + chemo ± bevacizumab (KEYNOTE-826)	PD-1 blockade + chemo + anti-VEGF	Phase III	1st line persistent/recurrent/metastatic CeCa	OS, PFS	Median OS 24.4 vs. 16.5 months; HR death 0.64 (PD-L1^+^); new standard
ISA101 + nivolumab	HPV16 peptide vaccine + PD-1 blockade	Phase II	HPV16^+^ tumors (mostly HNSCC, some CeCa)	ORR	ORR ∼33% overall; CeCa efficacy not established (101)
BMX-001	Mn porphyrin redox modulator	Early-phase (non-CeCa)	Glioblastoma, HNSCC	Safety, radiosens	Investigational; no CeCa-specific data
Mdivi-1	Mitochondrial division inhibitor	Preclinical	Cell/animal models	Mechanistic	Tool compound; not clinically viable
Metformin/Imipramine/Nelfinavir	Repurposed metabolic/redox mod	Preclinical/early phase	Cell/animal ± non-CeCa trials	Exploratory	CeCa-specific data absent; hypothesis-generating

Abbreviations: CeCa, Cervical Cancer; R/M, Recurrent/Metastatic; PD-1, Programmed Death-1; PD-L1^+^, Programmed Death Ligand-1, positive; ORR, Objective Response Rate; irAEs,Immune-related Adverse Events; Chemo, Chemotherapy; Anti-VEGF, Anti-Vascular Endothelial Growth Factor; OS, Overall Survival; PFS, Progression-Free Survival; HPV16^+^, Human Papillomavirus type 16 positive; HNSCC, Head and Neck Squamous Cell Carcinoma; Mn, Manganese.

### 5.4 Mechanistic rationale for combinations

Resistance to ICIs arises from multiple mechanisms, including activation of the adenosine/CD73 axis, compensatory upregulation of inhibitory receptors (LAG-3, TIM-3, TIGIT), and cancer-associated fibroblasts (CAFs)-CXCL12 signaling (96, 97). Preclinical studies suggest that redox normalization can reduce PD-L1 expression, restore antigen presentation, and enhance tumor immunogenicity (98, 99). These insights provide a rationale for combining redox-targeted therapies with ICIs (100). However, no patient-level CeCa data currently validates this approach, highlighting a key translational gap.

## 6 Future directions and conclusion

Despite significant progress in elucidating the molecular underpinnings of cervical cancer (CeCa), translation of mechanistic insights into clinical benefit remains limited. The interplay between HPV oncogenes, oxidative stress, and immune dysregulation presents both challenges and opportunities for therapeutic innovation. Several key priorities emerge for future research.

1. Development of predictive biomarkers. Reliable biomarkers are essential to stratify patients and guide therapy. While PD-L1 expressions and tumor-infiltrating lymphocytes (TILs) offer partial predictive value, their wider application is restricted following lack of multiplexed or composite biomarker strategies. Integration of oxidative stress markers (e.g., 8-OHdG, cf-mtDNA) with immune profiling may improve patient selection for immune checkpoint inhibitors (ICIs) and combination therapies. Nonetheless, standardization of assay platforms and pre-analytical workflows is critical for reproducibility and clinical translation.
2. Advancement of noninvasive monitoring. Liquid biopsy approaches, particularly cf-mtDNA and circulating immune signatures, represent promising tools for real-time monitoring of disease progression and therapeutic response. Rigorous prospective validation in large, clinically annotated cohorts is required before clinical adoption.
3. Rational therapeutic combinations. Evidence suggests that redox modulation may enhance immunotherapy efficacy by restoring antigen presentation and reducing PD-L1 expression. Rational design of trials integrating ICIs with redox-targeted agents, therapeutic HPV vaccines, or repurposed drugs could provide synergistic benefit. However, careful attention must be paid to potential antagonistic interactions and context-dependent effects.
4. Preclinical model refinement. Current *in vitro* and *in vivo* models incompletely recapitulate the complexity of the CeCa tumor microenvironment (TME). Integration of multi-omics platforms, single-cell technologies, and spatial profiling will be essential to capture the dynamic crosstalk between HPV-driven pathways, oxidative stress, and immune regulation. Such models will accelerate discovery of therapeutic vulnerabilities and facilitate translational research.

## 7 Conclusion

Cervical cancer remains a major global health challenge, with pathogenesis shaped by intricate interactions between HPV oncogenes, oxidative stress, and immune modulation. While immune checkpoint inhibitors and emerging redox-targeting strategies provide new therapeutic avenues, clinical benefit is limited to subsets of patients. Current evidence underscores the context-dependent duality of immune subsets such as Th17 cells and antioxidant enzymes like superoxide dismutase 2 (SOD2), reflecting the complexity of redox–immunity crosstalk.

Future progress hinges on the development of predictive biomarkers, noninvasive monitoring strategies, and rationally designed therapeutic combinations. Integration of multi-omics data with advanced preclinical models will be pivotal in bridging mechanistic insights into personalized treatment. By addressing these gaps, it may be possible to translate biological understanding into durable clinical benefit and improve outcomes for women with CeCa.
